# Nephron sparing surgery for a patient with a complicated solitary functioning kidney and a giant pT3 renal cell carcinoma: A case report

**DOI:** 10.3389/fsurg.2023.1094472

**Published:** 2023-03-17

**Authors:** Minghao Yu, Jiatong Zhou, Xun Shangguan, Subo Qian, Jie Ding, Jun Qi

**Affiliations:** Department of Urology, Xinhua Hospital Affiliated to Shanghai Jiaotong University School of Medicine, Shanghai, China

**Keywords:** solitary functioning kidney, nephron-sparing surgery, renal cell carcinoma, acute upper ureteral obstruction, case report, open nephrectomy

## Abstract

A solitary functioning kidney (SFK) with renal cell carcinoma (RCC) is an imperative indication for nephron-sparing surgery (NSS). Nevertheless, a giant pT3 RCC mass (maximum diameter >20 cm) on the functioning side of a patient with SFK is extremely rare. However, whether NSS is more beneficial than radical nephrectomy (RN) in such patients is controversial. Here, we present the case of a 71-year-old female patient with a 20 cm*16 cm RCC mass in the SFK, who initially presented with hematuria and acute urinary tract obstructive anuria caused by renal calculi. The patient underwent NSS treatment after our evaluation, and the 26-month follow-up revealed that her renal function recovered to the state before the tumor formation. In addition, no relapse or metastasis was detected.

## Introduction

Solitary functioning kidneys (SFK), whether congenital or acquired, are very common in the clinic. However, when malignant tumors occur in the unilaterally functioning kidney, the condition of patients with SFK becomes much more complicated. Providing proper management for these patients and improving the prognosis remains a knotty problem for urologists.

Nephron-sparing surgery (NSS) is the standard treatment for patients with tumors in the SFK. Although partial renal parenchyma has been reserved after NSS, the question of whether patients could achieve oncological control and renal function preservation depends on the tumor size, together with the presence of serosal infiltration, lymph node involvement, and distant metastasis. In addition, the surgical scheme, the skills of surgeons, and rational adjuvant therapy are also closely related to good long-term prospects. Owing to the difficulty of achieving total excision during the operation, tumors with a locally extensive size have a higher relapse or metastasis rate than small ones after NSS (1). Therefore, the National Comprehensive Cancer Network Clinical Practice Guidelines in Oncology (NCCN Guidelines, V1.2021) recommend only NSS for patients with stage pT3 when it is clinically indicated (2). However, both the American Urological Association Guidelines (AUA Guidelines, 2021) and the European Association of Urology Guidelines (EUA Guidelines, 2022) do not recommend NSS for patients with pT3 RCC (3). Selecting NSS or radical nephrectomy (RN) for patients with RCC in the SFK remains a matter of controversy.

Herein, we report the case of a patient with pT3aN0M0 renal cell carcinoma (RCC) in the SFK. The patient displayed symptoms of acute upper ureteral obstruction and was diagnosed with a 20 cm*16 cm malignant tumor mass in the right kidney by positron emission tomography-computed tomography (PET-CT) imaging, in addition to a pathological examination in Shanghai, China. After a multidisciplinary discussion, our surgery team implemented an NSS scheme on the patient that included open right partial nephrectomy, together with the removal of calculi, pyeloureteroplasty, and nephrostomy. The most recent renal function examination showed that the serum creatine (SCr) level, the blood urea nitrogen (BUN) level and the estimated glomerular filtration rate (eGFR) were close to those before RCC formation. So far, no serious surgical complications or acute renal injury (AKI) have occurred, and a postoperative follow-up revealed no metastasis. We present the following article under the CARE reporting checklist.

## Case presentation

**Figure 1:**
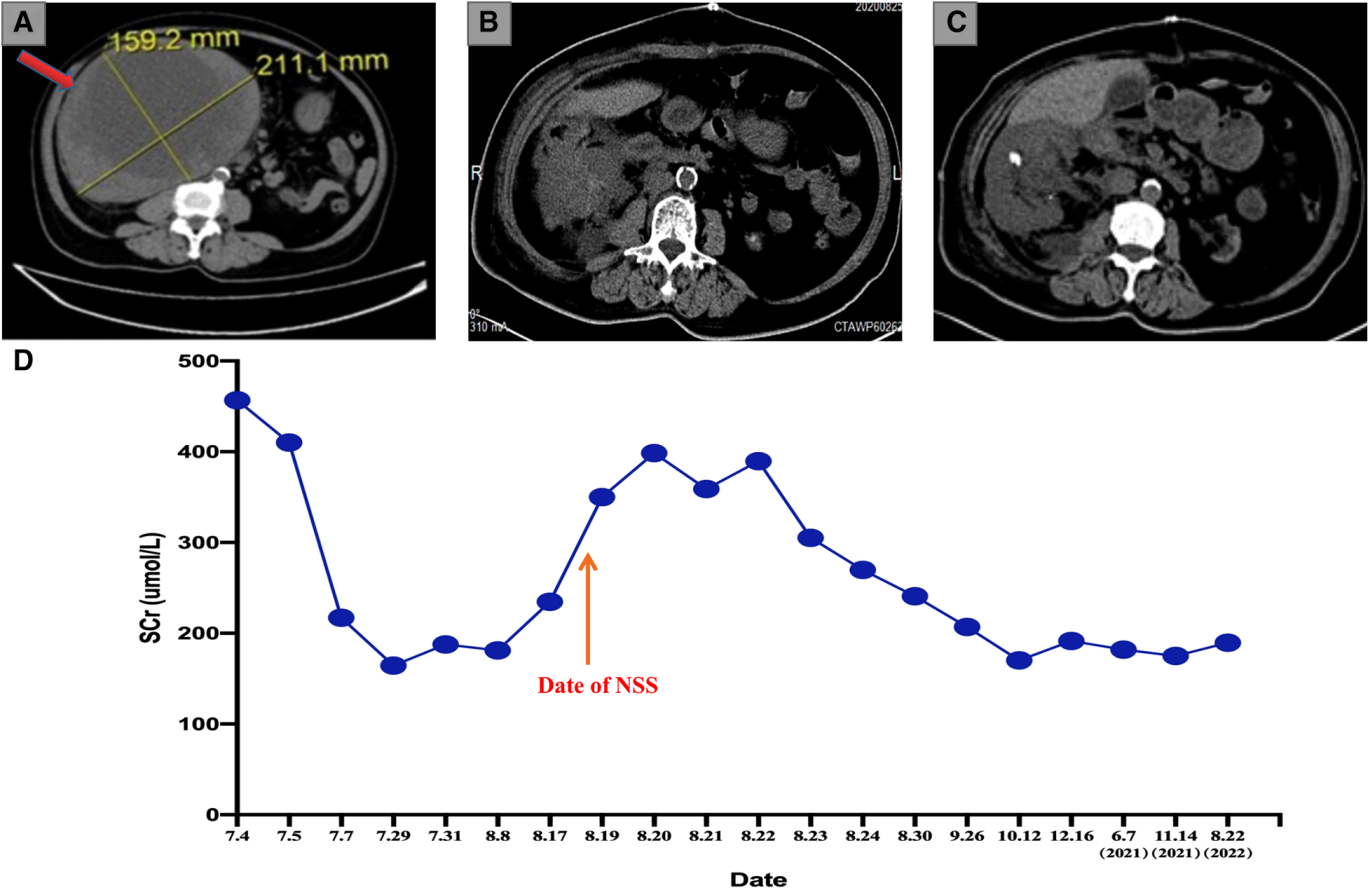
(**A**) Preoperative abdominal CT examination showing a 20 cm*16 cm RCC mass (the red arrow). (**B**) Abdominal CT scan after 1 week of the operation indicates a complete resection of the giant RCC mass. (**C**) The most recent abdominal CT scan indicates no sign of relapse. (**D**) The tendency chart presents the changes in the SCr level along with the progression of disease and treatment. CT, computed tomography; RCC, renal cell carcinoma; SCr, serum creatine.

**Figure 2:**
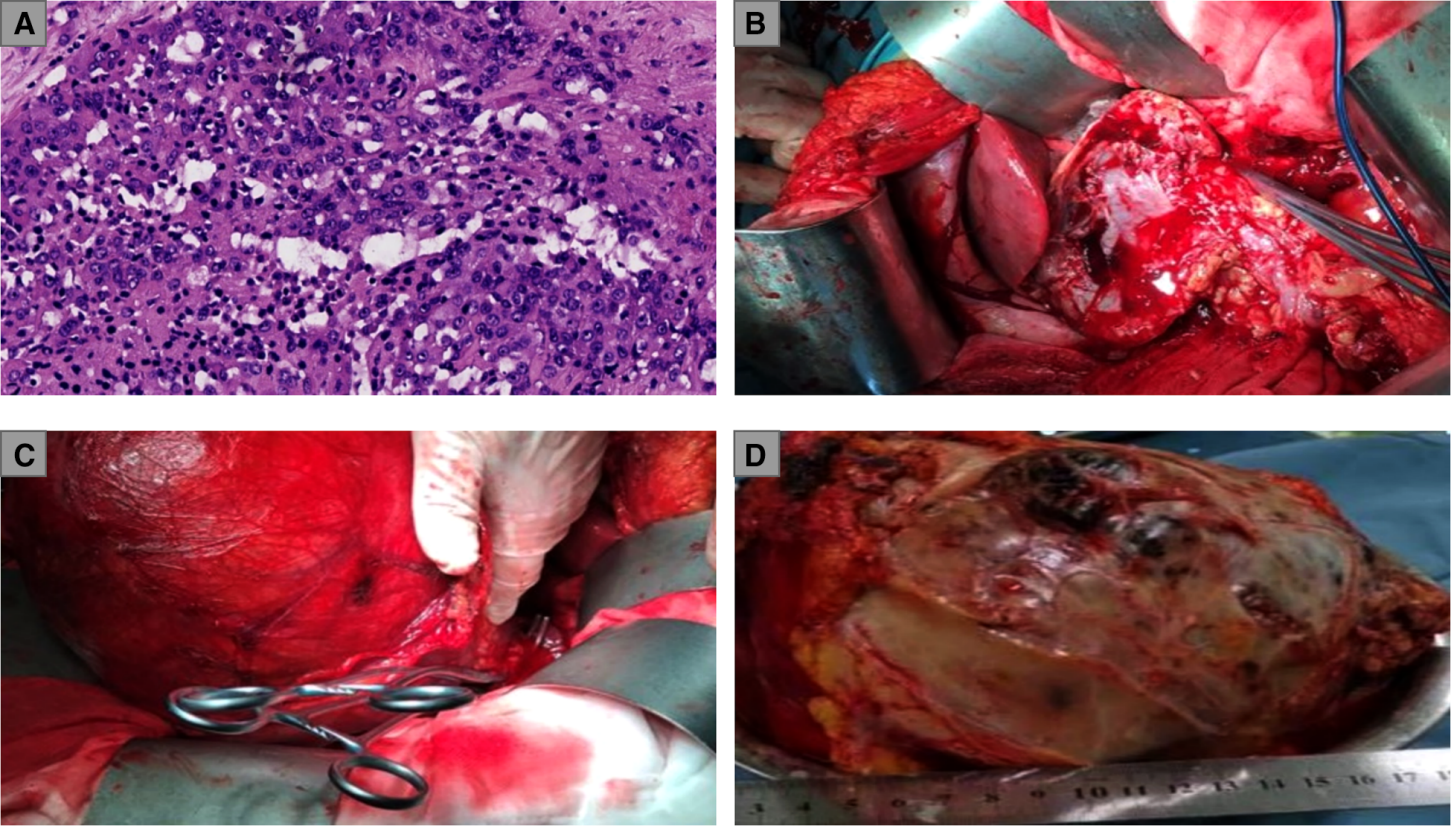
(**A**) Pathological examination of renal biopsies: renal adenocarcinoma, ISUP grade IV; (**B**) Exposure of the surgical field and the clamping of the renal artery; (**C**) A considerable part of the RCC mass was shifted out of the body for the convenience of surgical operations; (**D**) weighing and measurement of the RCC mass. ISUP, International Society of Urological Pathology; RCC, renal cell carcinoma.

A 71-year-old female was referred to the outpatient clinic of our hospital because of non-induced hematuria for 1 week and anuria for 1 day, accompanied by a fever (38.7°C) (4 July 2020). The patient was in poor health since she had suffered from hypertension and diabetes for many years. Her left kidney failed to function because of atrophy caused by unilateral renal calculi 20 years ago (eGFR of the left kidney: 2.64 ml/min/1.73 m^2^). Bilateral multiple renal calculi were found under a B-ultrasound. In addition, CT imaging of the abdomen showed a right renal mass (211 mm × 159 mm) but revealed no hemorrhagic foci around the mass or thrombi in the renal vein (Figure 1A). The SCr level indicated serious renal decompensation (456.7 umol/L). Since the right renal mass showed no signs of rupture, we initially attributed the hematuria and fever symptoms to the acute upper ureteral obstruction caused by renal calculi. In order to alleviate the symptoms of urinary tract obstruction and infection, we performed ureteroscopic lithotripsy and double-J stent placement in the right ureter along with anti-infection treatment (5 July 2020). However, the patient presented with gross hematuria, dysuria, and fever again only a few days after the double-J stent was removed (30 July 2020). A PET/CT scan revealed a space-occupying lesion with increased metabolism at the lower pole of the right kidney but no obvious metastasis in the whole body. Several enhancement patterns around the mass were also presented by the scan, and we considered them to be signs of hemorrhage (30 July 2020). The laboratory examination revealed that the SCr level was 187.6 μmol/L, and the BUN level was 19 mmol/L, which indicated that renal decompensation still existed. The renal function test showed that the eGFR of the right kidney was 23.9 ml/min/1.73 m^2^ (31 July 2020). To study the characteristics of the giant mass, an ultrasound-guided renal biopsy was performed, and pathology reported RCC [renal adenocarcinoma, grade IV according to the International Society of Urological Pathology (ISUP) grading system, with transcription factor EB (TFEB) amplification] (31 July 2020) (Figure 2A). As the patient and her family requested to preserve renal function, our surgical team decided to perform NSS on the patient after a discussion, using RN and emergency hemodialysis as alternate schemes.

**Figure 3:**
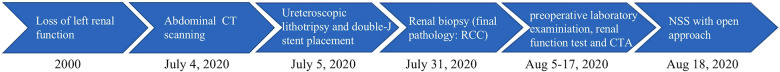
Timeline of the medical history, examination, and treatment.

Considering the large size of the renal mass, our surgical team settled on an open partial nephrectomy (OPN) to remove it. The operation was scheduled 18 days after the biopsy (18 August 2020). We prescribed Amlodipine Besylate and Novolin to control blood pressure and blood glucose, in addition to Aztreonam for anti-infection before the operation. The CT angiography (CTA) presented the origin of the extrarenal arterial blood supply of the tumor mass but revealed no vascular abnormalities in the functioning kidney (5 August 2020). The preoperative dialysis was performed to depress the SCr level, and the SCr level was 234.6 umol/L before the operation (17 August 2020). In addition, steps were taken to ensure that the liver and kidney functioned normally so that the patient could tolerate the surgery. During the operation, an L-shaped incision was made by the surgeon in the right upper abdomen, finding the giant RCC mass on the ventral side of the lower right kidney with ureteral dilatation. The surgeon completed the dissection of the perirenal adipose tissue in order to expose the tumor mass and shifted a considerable part of the mass out of the body for the convenience of surgical operations. In addition to the tumor resection, the surgeon removed the calculi in the upper segment of the renal pelvis and ureter and a part of the renal calyces and then performed the right pyeloureteroplasty procedure to help maintain renal function and a nephrostomy to facilitate postoperative drainage. The renal artery was clamped during the mass resection, and the total ischemia time was 110 min. Thus, our surgical team placed ice crumbles around the surgical field to reduce the renal surface temperature and preserve renal function. The total bleeding volume was 800 mL, and the transfused red blood cells (RBCs) and plasma levels were 4U and 400 mL, respectively. The resected mass weighed 5.8 kg (Figures 2B–D). A postoperative abdominal CT scan (August 25, 2020) indicated the total resection of the RCC mass (Figure 1B), and a pathological examination of the RCC mass showed that the renal sinus adipose tissue was involved locally, with no obvious breakthrough of fibrous capsule and no tumor involvement in the renal cutting edge or perirenal adipose tissue (pT3aM0N0, UISS III). After nearly 2 weeks of the surgery, the result of a renal function examination revealed that the SCr level was 240.8 μmol/L and the BUN level was 10 mmol/L (30 August 2020). The patient did not take any medicine after NSS and was followed up every 3 months. Now, 26 months have passed since the operation, and the latest chest and abdominal CT scanning showed no sign of relapse or metastasis (June 3, 2022) (Figure 1C). In addition, the most recent follow-up revealed that the SCr level was 189.5 umol/L (Figure 1D), the BUN was 17.74 mmol/L, and the eGFR of the right kidney was 30.3 ml/min/1.73 m^2^ (22 August 2022). The complete timeline is shown in Figure 3.

## Discussion

SFK refers to a condition in which patients have two kidneys anatomically but have to rely on one to support normal physiological activity since the other one has failed to function. Acquired SFK usually results from unilateral renal trauma or severe calculus obstruction. When the acquired SFK is complicated with RCC, the tumor mass on the functioning side always leads to renal failure. Thus, for those patients with acquired SFK and RCC in whom surgery is indicated, a timely resection of the tumor mass is vital for saving their lives. Choosing the appropriate type of operation is the key to reliable oncological control and decreasing the probability of postoperative complications for these patients.

SFK is considered the imperative indication for NSS, while NSS is not recommended for pT3/pT4 RCC patients (2, 3). For patients with pT3 RCC, most guidelines recommend RN for tumor resection. When surgeons deal with pT3 RCC in patients with SFK, the question of which type of treatment, NSS or RN, could benefit patients more begs an answer. The major advantage of NSS for patients with SFK is to preserve a partially functioning kidney and prevent them from submitting to renal replacement therapy or renal transplantation. In addition, accumulating evidence supports that NSS could achieve similar overall survival (OS) and cancer-specific survival (CSS) rates with RN, and the preservation of renal function helps patients improve their quality of life after the operation. Moreover, NSS significantly reduces the incidence of long-term cardiovascular accidents and chronic kidney disease (CKD). However, performing NSS on patients with pT3 RCC is a great challenge for surgeons, as the radical resection of RCC masses is difficult to perform during NSS. Studies have shown that for patients with pT2-pT4 RCC, margin positive rates are higher in those who receive NSS than in those who receive RN. Due to the relatively long operation and ischemia time, patients with a large-volume RCC who undergo NSS are prone to having short-term complications (4–6). In summary, we can conclude from the above comparison that NSS and RN have their own advantages and disadvantages in dealing with patients with pT3 RCC. In terms of the surgical approach, laparoscopic nephrectomy has been proven to have many advantages, while we chose OPN for our patient because of its ease of operation and to avoid tumor rupture during resection. No serious complication occurred after the OPN procedure, which proves that OPN is still a good choice when handling locally complicated and large RCCs.

Preoperative examination and treatment are indispensable steps in the process of resection. A general examination should be carried out to check whether general health conditions such as blood pressure, cardiac function, pulmonary function, and so on enable patients to withstand surgery. Adjustments for comorbidities in addition to abnormal bleeding and coagulation function are necessary. For patients with poor general conditions, NSS is not recommended (7). The preoperative eGFR and Scr levels need to be evaluated to check renal function before surgery. The abdominal CT scan and CTA assist surgeons to assess the tumor complexity and uncover aberrant vessels, which are crucial for the evaluation of surgical complexity and outcomes. Clinical trials of neoadjuvant TKI-drug therapy found that high-risk RCC patients (pT3, pT4, and N+) enjoyed disease-free survival (DFS) benefits and a reduction in tumor volume (8). The results indicated that preoperative neoadjuvant therapy with targeted drugs could help some patients switch from RN to NSS. The patient in our case did not receive neoadjuvant therapy before the operation. Owing to the symptoms of acute ureteral obstruction and the huge volume of RCC mass, urgent surgery was required, and hence, we dealt with only her basic disease condition and decreased the SCr level.

Surgeons might choose RN to ensure the total resection of RCC masses from patients with pT3 RCC in an SFK, which will reduce the risk of tumor relapse. However, the most prominent disadvantage of this surgical choice is that patients need to receive renal transplantation or long-term hemodialysis after RN. This will severely affect their daily lives and place a huge economic burden on their families. Therefore, in addition to a comprehensive evaluation of the patient and tumor, the opinions of the patient and their family are also very important when choosing the type of surgery. Before a decision is made on the surgical scheme, surgeons should fully communicate with patients and their families and honor the wishes of patients. Our patient and her family strongly requested that we preserve the functioning kidney, and in response, we carefully considered the opinions of the patient and her family before making the decision.

We have summarized some key points that were worth noting during NSS. Before blocking the renal artery, the size and firmness of the artery clamp should be checked in order to avoid incomplete blocking of vessels, which might cause intraoperative bleeding. The surgeon has to mark the resection range of the tumor mass along the edge of the normal renal tissue to make sure that the mass or the pseudocapsule is not ruptured, and then, carefully cut approximately 0.5 cm–1 cm away from the edge of the mass to maintain the integrity of the tumor capsule, which is significant for complete excision and preventing tumor rupture during NSS. It is widely recognized that a short ischemia time is crucial for preserving renal function after the operation. Previous research suggested that human kidneys could tolerate 30–60 min of controlled clamp ischemia with minor damage renal parenchyma and no acute renal function loss. However, Bravi et al. reported that a distinct difference existed in the risk of AKI between patients who had an ischemia time <10 min vs. >20 min (9). In our patient, the ischemia time was much longer than 60 min. Nevertheless, the patient still successfully preserved her renal function, and AKI did not occur after the operation. We believe that the ice in the surgical field greatly helped in preserving the renal parenchyma to the maximum extent. Along with the experience from other NSS cases, we suggest that surgeons lower the temperature of the surgical field to approximately 15–0°C, which could preserve the renal function from being damaged within 2 h. Postoperatively, the exact suture of the wound is critical for preventing bleeding. As OPN always results in deep wounds, a multilayer suture is recommended. We hope our tips could provide some reference for peers who handle similar cases.

Regular postoperative follow-up could help surgeons detect tumor recurrence, metastasis, renal insufficiency, and other problems on time and deal with them immediately. The follow-up should include renal function tests, a chest and abdominal CT examination, and laboratory examinations that measure the serum parameter of renal function. The patient reported in our case was followed up every 3 months, and the chest and abdominal CT examination revealed no signs of relapse or metastasis to date.

## Conclusions

Our case provides evidence that NSS is a reasonable option for SFK patients with a local pT3 RCC. We believe that a comprehensive evaluation of the patient’s general condition, renal function, and tumor complexity is crucial for the formulation of the surgical plan. In addition, there are many important points to consider during NSS for the preservation of postoperative renal function and the prevention of perioperative complications. We hope to share our experience with our peers who handle similar cases.

## Data Availability

The original contributions presented in the study are included in the article/Supplementary Material; further inquiries can be directed to the corresponding author.
